# Modern Compact Cities: How Much Greenery Do We Need?

**DOI:** 10.3390/ijerph15102180

**Published:** 2018-10-05

**Authors:** Alessio Russo, Giuseppe T. Cirella

**Affiliations:** 1 Department of Landscape Design and Sustainable Ecosystems, Peoples' Friendship University of Russia, RUDN University, 117198 Moscow, Russia; alessio.landscape@gmail.com; 2 Polo Centre of Sustainability, 18100 Imperia, Italy; 3 Faculty of Economics, University of Gdansk, 81-824 Sopot, Poland

**Keywords:** garden cities, ecosystem services, healing garden design, biophilic urbanism, edible green infrastructure

## Abstract

The modern compact city is identified as a high-density and mixed-use pattern. Its features are believed to contribute to a form of functional urban design that supports sustainability and, restresses, the importance of ecosystem services. Urban green space (UGS) plays a vital role in the design and impact on how compact cities have developed and triggered a scientific discord on the amount of greenery individuals require and to what extent contemporary approaches address the question. Research points to at least 9 m^2^ of green space per individual with an ideal UGS value of 50 m^2^ per capita. An examination on the perception, use, quality, accessibility and health risks of urban green and blue spaces is explored, alongside the availability of novel UGS and greenery-related approaches that investigate compact city design and planning for health and wellbeing. The amount of ‘green’ and relating UGS availability in cities indicates vital knowledge modern compact cities must consider.

## 1. Introduction

At present, cities are facing a number of environmental issues which influence the wellbeing and livelihood of millions worldwide. The consequences of urban sprawl have resulted in pollution, consumption of resources and energy and various types of dumping grounds [1]. Urbanisation and densification processes have led to a loss of urban green space (UGS) and biodiversity within cities, particularly within Asia and Australia and to a lesser extent Europe and North America [2]. With the increasing frequency and severity of environmental hazards and climate change such as heat, urban design strategies will play an important role in reducing vulnerability, promoting health and building resilience [3]. In response to these environmental hazards and urban densification (i.e., via rapid population growth and rural to urban migration), there is a growing need to innovate healthier designs and planned sustainability for resilient urban environments.

The compact city is identified as a high-density and mixed-use pattern which leaves space for the countryside, husbandry, nature and recreation. It includes a well-ordered distinction between the city and countryside (i.e., a ‘counterbalance’) in physical appearance and land use functions to city-dwellers [4]. This type of practice is believed to restrain urban sprawl by intensifying activity in urban dense regions, reducing personal vehicle trips and providing diverse services through mixed land use and revitalisation of old urban areas (i.e., discouraging infill development) [5,6]. These features are believed to contribute to a form of functional urban design that, in turn, would support a more sustainable living relationship within such environments [6]; however, compact cities that have an overall lower percentage of UGS demonstrate to lack ecosystem services [7]. Moreover, such cities are the most impacted by the heat island effect [8] and the resulting consequences from urban densification. Densification has shown to be largely unhealthy within these urban neighbourhoods affecting mostly local residents; the permanence of urban built structures makes it likely future residents’ health will also be affected by conditions resulting from today’s urban planning which is not restricted to the short term [9,10]. In order to contrast the urban heat island and effectively provide ecosystem services, sufficient and high-quality UGS and or other greenery elements should be readily made available to urban residents. Improving public health through urban development and greenery renewal of compact cities is an important part of the sustainable development concept [9]. 

Local authorities, increasingly, are in search of new, adaptive and flexible forms of urban gardening, characterised by high accessibility and hybrid functions [11]. As a result, a number of relating inferences have been made regarding urban allotment garden space and newly implemented urban gardening infrastructure [12]. Research indicates urban gardening practices that are desirable, smart and compact in form and function support sustainable practices, increase liveability and higher urbanised standards of development [11,12]. Tappert et al. [11] state traditional forms such as urban allotment gardens have been problematised as seemingly incompatible with the requirements of UGS provision in the overall platform of compact cities. In this paper, we elaborate upon this discussion by examining quality, accessibility, health risks of UGS, attitudes toward UGS and how landscape architects and urban planners can incorporate greenery via the delivery system of ecosystem services to inhabitants for more resilient and healthy compact cities. Furthermore, it highlights the amount of greenery needed by interconnecting concepts within a compact city garden approach.

## 2. Urban Green and Blue Space: Perception, Use, Quality, Accessibility and Health Risks

With the maturation of modern compact cities, urban green and blue space (UGBS) inquiries has become an integral part of urban green infrastructure (UGI) [13]; nonetheless, there is evidence UGBS benefits are not equitably distributed across diverse cities and urban populations [14]. Understanding the relationship between urban population and quality and amount of green space is vital in terms of sustainability, health and resilience of urban areas. According to Hunter and Luck [15], UGBS offer a variety of social qualities that focalise on anthropogenic attributes directly influencing its ‘green’ status in terms of accessibility, recreational use (i.e., visitation rates and leisure activities), management as well as political and financial benefits. 

In terms of the ecological quality of UGBS, the natural attributes of green space comprise of plant and animal diversity and abundance, flower density and tree canopy cover [15]. Blue space adaptation and infrastructure are associated with new and existing mixed-use urban development and specifications relating to urban heat-stress and spatial- and scenario-based planning. It has been shown that visitor perception can influence city patterns (i.e., its urban layout) and inform planners and management of effective urban designs [16]. Good quality UGBS improves quality of city life via the enhancement of their attractiveness to residents, employees, tourists, investors and firms [17]. From a social perspective, UGBS has an impact on a wide range of issues ranging from community involvement and empowerment, including matters of safety, inclusion, equality, civic pride, health, education and recreation. Well managed and maintained UGBS can contribute to social inclusion and social justice and provide cultural links and opportunities for community events and outdoor activities [18]. However, the majority of UGBS are limited in size, occluded within the built-up matrix and separated from each other by harsh and often inhospitable developed areas [19]. People who find UGBS attractive, pleasant and safe are more likely to use them, whereas those, especially females, who feel unsafe will tend to avoid them entirely [16]. While there is a growing body of evidence suggesting that contact with nature, often referred to as UGBS is associated with multiple health benefits and wellbeing impacts [20], it may also have potentially negative effects (i.e., ecosystem disservices) and health risks [13,21]. Thus, lack of planning in the design and management of urban spaces and in the choice of ornamental species has been among the factors triggering pollen allergy which is one of the most widespread diseases in urban populations [22]. The selection of species is very important for the quality of the air in our cities, for examples high Biogenic Volatile Organic Compounds (BVOC) emitter trees might contribute to ozone formation [23]. Moreover, when BVOCs from UGBS occur in urban areas with high human population densities (e.g., compact cities), they can have much greater health damages than those from natural forests [24].

Additionally, people using UGBS could potentially come into contact with *Borrelia* infected ticks [25]. Without research-based knowledge and public support, UGBS could fail to meet community needs and attract undesirable elements or activities and, in extreme cases, be utterly abandoned by users [26,27]. Poor design of UGBS can produce several significant social costs and setbacks [28] (Table 1).

Scientific literature has mostly focused on approaches that examine ecosystem services provision, perception, use and quality of UGBS. Three Eurocentric perspectives illustrate notable examples. First, Bertram and Rehdanz [29] analysed cultural ecosystem services provided by urban parks in four European cities. They compared attitudes toward ecosystem services provision, perception and use of urban parks by investigating park visitors. Results indicated similarities between cities and the importance of different park characteristics. Second, Arnberger and Eder [30] developed a conceptual framework of integrating physical and social characteristics of different green spaces throughout Vienna, Austria by examining preferences of 692 on-site visitors. They found visitors, generally, preferred green space when seeking stress relief. Third, Natural England [31] developed the ‘Accessible Natural Greenspace Standard’ for England which recommends everyone, wherever they live, should have accessible green space. These guidelines point out the amount of accessible green space for each individual. On the other hand, ‘formal greenspaces’ (e.g., parks and residential gardens or yards) may not be sufficient to meet some residents’ needs, especially in denser environments [32,33]. To this end, Rupprecht et al. [33] have examined how residents perceived and used informal green spaces (IGS) (i.e., vacant lots, street or railway verges and riverbanks) in Brisbane, Australia and Sapporo, Japan. They found that respondents in both cities knew, appreciated and used IGS in their neighbourhood and were attracted by its proximity, natural features and absence of use restrictions but also valued a certain level of human influence. In terms of accessibility, it was found that Brisbane’s IGS levels were 78 % accessible, 7 % partially accessible and 15 % not accessible, while in Sapporo it was slightly different with 68 %, 21 % and 11 %, respectively [34].

We have determined that perception, use, quality and accessibility of UGBS and also IGS play a dynamic part in educating planners and intelligentsia by way of knowledge and fruitful design of modern compact cities. Ensuring human comfort, by designers and landscape architects alike, the use of green areas and a cyclic process of rethinking via the exemplification of reinvention, transformation, perception and evaluation is required.

## 3. Green Space Availability and Novel Approaches for the Design and Planning of Compact Cities

UGS availability, to date, historically relates to the chronology of events and activities that have occurred and decisions that have been formulated over the past few centuries; from a historical perspective, urban complexities have spotlighted a number of key indicators that have propelled improvements in human health, wellbeing and socio-ecological interactions within urban environments (e.g., urban centres providing a per-capita threshold value for UGS or implementing a minimum distance to available green space) [35]. The World Health Organization [36] recommended the availability of a minimum of 9 m^2^ of green space per individual with an ideal UGS value of 50 m^2^ per capita. These statistical values correlate with a number of UGS standards, including: (1) linkages between sustainable cities and better health, (2) core health indicators to monitor progress and identify success, (3) expanding indicators values (e.g., governance indicators, access to health and sanitation services, food markets and urban infrastructure for social, recreation and livelihoods), (4) adding value to health indicators and (5) feasibility of data reporting via cross-cutting issues (e.g., equity, governance and climate change). In retrospect to the amount of greenery and relating UGS availability in cities and urban areas we delineate a linkage between the World Health Organization’s UGS values with a reduced amount of social and environmental discontent [37,38,39,40].

**Figure 1:**
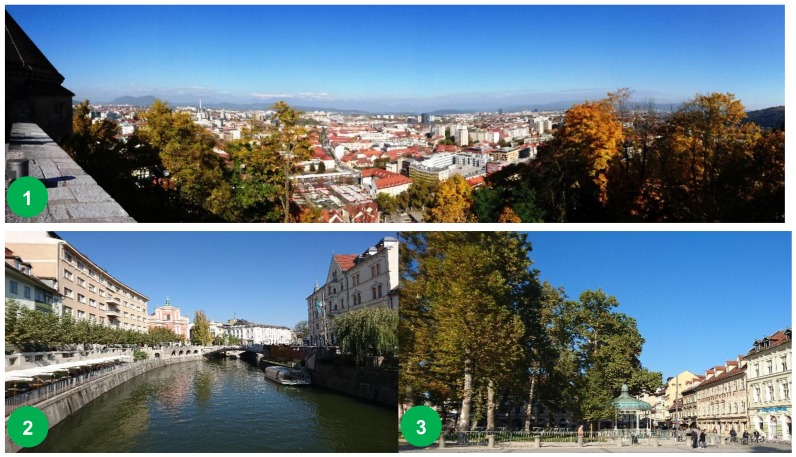
Ljubljana, Slovenia 2016 European Green Capital: (**1**) Panoramic view of the city’s vegetation, (**2**) Vegetation along canals, (**3**) Large trees and canopy cover (Photographs taken by G. T. Cirella, 22 October 2017).

An example of an ideal compact city is Ljubljana, Slovenia, awarded the 2016 European Green Capital, in which almost 560 m^2^ of UGS is available per inhabitant and virtually all its residential zones lie within a 300 m radius from public green space [41]. Over the past two decades, Ljubljana’s transformation—via urban planning, landscape architectural provision and sustainability thinking—has significantly propelled it from its Socialist past toward a modern ‘green,’ compact city (Figure 1). This emphasis on UGS policy has focalised the city on restorative and conservation-leaning development. Urban development, in the context of sustaining city compactness, is directed primarily on regeneration and renewal of existing developed areas and the rehabilitation of degraded ones [42]. At present, Ljubljana’s high level of environmental awareness has it as one of the world's most sustainable cities, ranking in the top 100 for the third time. 

Correspondingly, Badiu et al. [43] maintain UGS per capita is not the target but the process, in which urban green planners can focus on developing UGI-related models that are adaptable to varying urban areas. A major challenge in urban design and urban planning approaches to health promotion is the difficulties associated with modifying existing environments (e.g., limited spaces for tree plantation) [44]. However, based on the literature, there is a growing amount of information that supports alternative planning and planning-based approaches that detail compact urban centres that exclusively advocate habitat-friendly areas. Example concepts for contemporary city planning include: UGI, nature-based solutions, biophilic urbanism, sponge cities (e.g., Shanghai and Wuhan in China) [45], forest cities, edible green infrastructure, eco-urbanism and landscape urbanism. 

**Figure 2:**
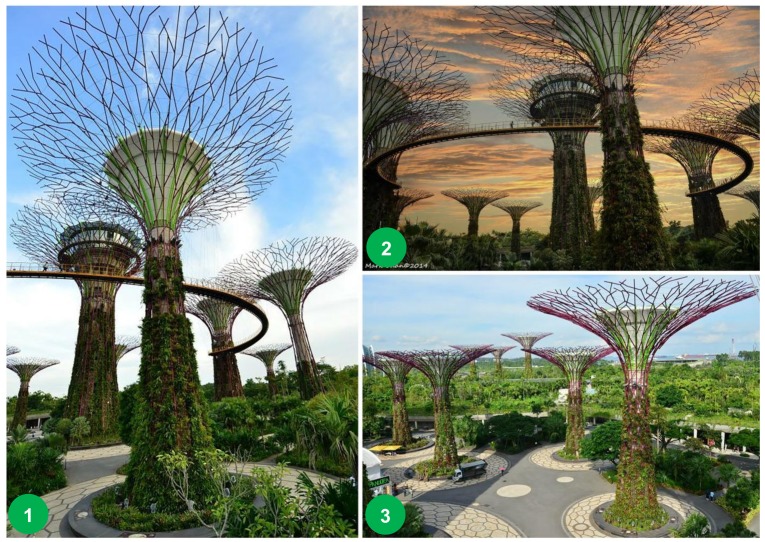
Singapore ‘Gardens by the Bay’: (**1**) Close up of the ‘Supertree Grove’, (**2**) Panoramic sunset view of the elevated walkway called OCBC Skyway, (**3**) Walking environment and scale of the gardens (Photographs taken by Mark Chan, 2014–2015).

UGI and nature-based solutions are terms that are frequently applied interchangeably; they both integrate natural systems with build systems and are often reported as having a key role in achieving a future-oriented compact city framework—both for liveability and sustainability [7,46]. The city of Singapore is often reported as an example of successful biophilic urbanism in which a shift in vision from a ‘garden city’ to a ‘city in a garden’ has slowly been developed over the past few decades [47,48]. An example of this development is the visionary project, with estimated costs of USD 750 million, in and around the Marina Bay Sands area—referred to as the ‘Gardens by the Bay.’ It features extraordinary nature-based systems built with a regenerative design of reclaiming foreshore and natural aesthetic beauty [47]. The project brings to life Singapore’s National Parks Board Service of creating a ‘city in a garden’ approach with iconic features, including the landmark ‘Supertree Grove’ in which tree-like vertical gardens, measuring between 25 and 50 meters tall, have been designed with large canopies that provide shade in the day and come alive with an exhilarating display of light and sound at night (Figure 2) [49]. 

**Figure 3:**
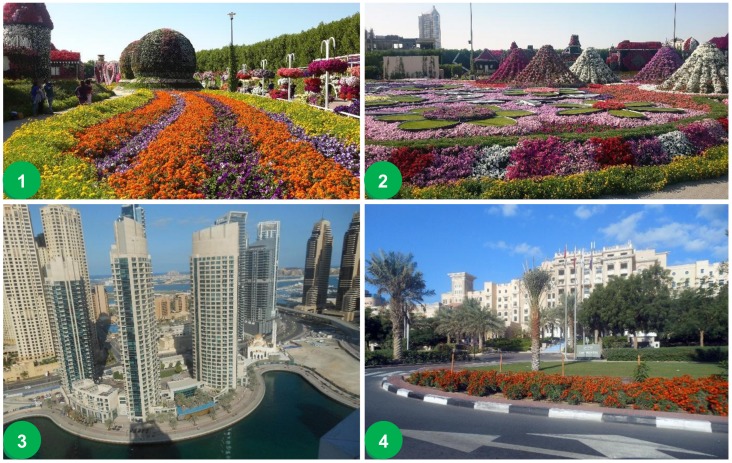
Dubai, UAE: (**1**) Dubai Miracle Garden with surrounding ‘green’ wall barrier in background, (**2**) Dubai Miracle Garden with integrated shading structures, (**3**) Dubai Marina with high-rise buildings and green rooftops, (**4**) Dubai Streetscape (Photographs taken by A. Russo, 13 November 2015).

Another example is Chicago, due to a billion-dollar investment since 2001, in which a wide range of projects, including the creation of new gardens and natural areas like Millennium Park and Palmisano Nature Park (i.e., a twenty-seven acre park created from an old stone quarry in the South Side Bridgeport neighbourhood) have revamped the greenery and UGS city-wide [50]. Conversely, biophilic urbanism takes into account the integration of building systems that focus on sustainable vegetation practices (e.g., by managing water and energy consumption) as well as design, installation and maintenance costs [51]. The re-naturing processes of cities with non-native species often can have a damaging effect (i.e., ecosystem disservices) and, correspondingly, high management costs. An example of this unsustainable course of action is the city of Dubai, UAE (United Arab Emirates), a water scarce area, which fronts exceedingly high maintenance costs and irrigation requirements (Figure 3). A policy intervention that considers alternative ecologically oriented design is urgently required in much of the Middle East. One failed example, is a lack of policy for landscape designers in utilising native plant species [52]. To our knowledge, Abu Dhabi, UAE, is the only major Middle Eastern city focalising on such regulations by way of its innovative green standards. Its design methodology, named ‘Estidama,’ the Arabic word for sustainability, constructs and operates buildings and communities by imposing building codes that are green-friendly, while still recognising its unique regional needs and expansive demands [53]. 

**Figure 4:**
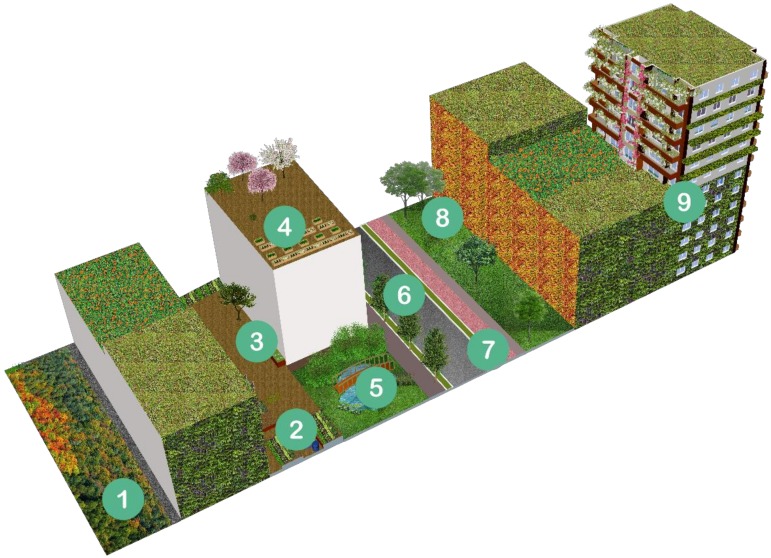
Landscape architectural designs and features of compact cities, promoting ecosystem services, biodiversity, mental health and wellbeing, include: (**1**) urban forest/urban parks, (**2**) allotment gardens, (**3**) vegetable raingardens, (**4**) edible green roofs, (**5**) detention and retention ponds/wildlife ponds, (**6**) street trees, (**7**) bioswales, (**8**) domestic/rain gardens, (**9**) building integrated vegetation (e.g., biodiverse green roofs, green walls and climbing plants).

Using a combination of approaches and information (e.g., sustainable design guidelines, biophilic design approach, IUCN (International Union For Conservation Of Nature) Best Practices Guidelines, UNESCO (United Nations Educational Scientific And Cultural Organization) Man and Biosphere Programme (MAB) and healing garden design approach [54,55,56,57,58]), cities can be regenerated by fashioning the thesis of ‘city in a garden’ to enrich ecosystem services, wellbeing and mental health (Figure 4). 

**Figure 5:**
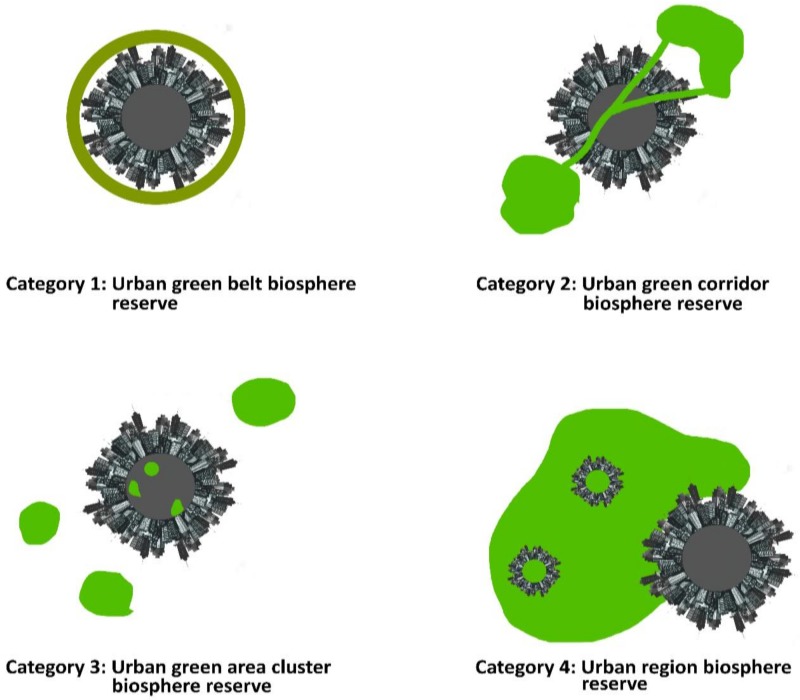
Types of biosphere reserves (Source: adapted from the 2018 UNESCO Man and Biosphere Programme, city image designed using ikatod|Freepik).

For example, the IUCN Best Practices Guidelines help to infuse nature into the built environment and break down the cultural barriers between ‘nature’ and ‘urban.’ According to these guidelines, there are three different ways of incorporating nature into the larger urban picture, this can be done via: (1) comprehensive, interdisciplinary scientific studies; (2) comprehensive local biodiversity strategies (e.g., Cape Town Biodiversity Strategy and Connecting with London’s Nature: The Mayor’s Biodiversity Strategy); and (3) region-wide coalitions (e.g., Chicago Wilderness and the London Biodiversity Partnership) [54]. The ecosystem approach, developed by UNESCO MAB, defines a strategy for the integrated management of land, water and living resources; it promotes conservation and sustainable use of resources in an equitable way [58,59]. In the Asia-Pacific, the principles of the ecosystem approach has been applied to a green rooftop in Seoul, Korea which (1) created a set of goals in securing green areas and biotopes in the downtown area, (2) created an urban eco-network, (3) procured a base for urban ecosystem study and environmental education and (4) disseminated ideas of coexistence between nature and a variety of subsets (i.e., humankind, wetlands, meadows, scrubs and woodlands, wall revegetation and a vegetable field) [59]. The UNESCO MAB working group identified four different categories of urban biosphere reserves (Figure 5) [58], in which Dogse [57] characterised it as “important urban areas within or adjacent to its boundaries where the natural, socioeconomic and cultural environments are shaped by urban influences and pressures and set up and managed to mitigate these pressures for improved urban and regional sustainability”.

**Figure 6:**
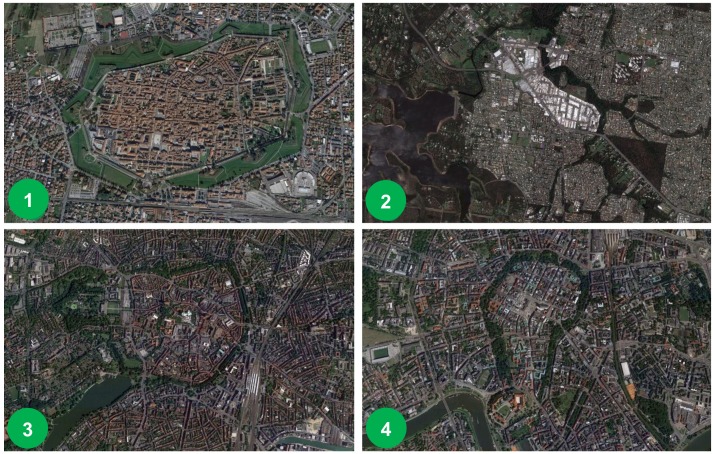
Examples of urban green belt biosphere reserves: (**1**) Lucca, Italy; (**2**) Capalaba, Australia; (**3**) Munster, Germany; (**4**) Cracow’s Old Town, Poland (Source: aerial views from Google Earth).

Dogse [57] is describing Category 1: Urban green belt biosphere reserve in Figure 4 in which a circular ring around a central urban area is depicted; some example cities include: Lucca, Italy; Capalaba, Australia; Munster, Germany; and Cracow’s Old Town, Poland (Figure 6). The remaining three categories in Figure 5 exemplify the varying options researched via UNESCO MAB and illustrate the capacious possibilities of UGS integration and approaches for the design and planning of compact cities. 

Future compact cities should also consider food insecurity and hunger. To this end, edible green infrastructure is a novel approach for the design of “edible cities” that have the potential to improve resilience and quality of life in cities as well as prevent food insecurity. Edible green infrastructure is a sustainable planned network of edible food components and structures within the urban ecosystem which are managed and designed to provide primarily provisioning ecosystem services. Typologies are based upon one macro category (i.e., edible green infrastructure and urban agriculture) as well as eight sub-classifications: (1) edible urban forests and edible urban greening, (2) edible forest gardens, (3) historic gardens and parks and botanic gardens, (4) school gardens, (5) allotment gardens and community gardens, (6) domestic and home gardens, (7) edible green roofs and vegetable rain gardens and (8) edible green walls and facades [13]. 

A new concept of vertical forests is gaining popularity as it promotes the coexistence of architecture and nature in urban areas [60,61]. Recently in the Asia-Pacific, a futuristic approach of forest cities has been proposed to deal with air pollution problems in China, the prototype of a new generation of small, compact and green cities, composed by dozens of tall and middle size buildings—the so called “Vertical Forests”—all surrounded by the leaves of trees (ranging from 3–9 m in height), shrubs and flowering plants [62]. Moreover, the vertical forests concept promotes biodiversity in cities since buildings are designed to be inhabited not only by humans but also by birds and insects. This regenerative practice takes place without the implication of expanding the city upon territory [61]. 

Again, the Asia-Pacific region has developed a notable project that is under development this year Tengah, Singapore; it is the first “Forest Town” that is fully integrated with its surrounding ecosystem with greenery formations as the main structural foundation of the town. Tengah will offer 42,000 residential dwelling units within five distinct districts: (1) Garden District, (2) Park District, (3) Brickland District, (4) Forest Hill and (5) Plantation District (i.e., home for the community farming). The key attraction will be an approximately 100 meter-wide forest corridor which will provide ample space for residents to enjoy nature and relating nature-based services. A large central park will serve as the ‘green lung’ for Tengah. The park will be integrated with ponds and canals, providing lush greenery and recreational promenades [63].

Another novel concept is the healing garden design approach used by Lau and Yang [64] in which a compact university campus in Hong Kong was built; its design enhanced health benefits and has produced a healthier university environment. A healing garden is ‘‘a garden in a healing setting designed to make people feel better’’ [65]. This approach can also be applied at the city-scale. The applicability of the SITES Rating System and specifically its site-specific performance benchmarks support the application of this approach, on the basis of: (1) using the concept of ecosystem services; (2) understanding natural processes; (3) uniting interdisciplinary best practices in landscape architecture, ecological restoration and related fields; and (4) acquiring knowledge-base through scientific literature, case-study precedents and SITES pilot projects [56,66]. Table 2 includes several strategies for the design of a ‘city in a garden,’ which takes into consideration ecosystem disservices [67,68,69,70,71]. 

By achieving these benchmarks, UGS site-specific performance in collaboration with the maintenance, support and enhancement of natural systems has proven to be an emerging indicator-based approach for the design and planning of compact cities. Well planned, maintained and designed compact cities have the potential to be both an environmentally sustainable and a liveable option [5]. However, the modern green city vision seems to make room only for park space, waterfront cafes and luxury ‘Leadership in Energy and Environmental Design’ certified buildings, prompting fear that there is no space for “sustainable” urban centres inclusive of industrial usages and the working class [74]. This modern vision can lead to negative social effects, for example, eco-gentrification can arise even when the primary motive in UGS provision addresses environmental injustice in its distribution [75]. Hence, the use of IGS has been proposed as an anti-gentrification strategy [76]. Furthermore, IGS is an emerging topic in urban greening research and plays a valuable role in providing a number of ecological and sociological benefits for urban residents [77,78]. IGS has no monetary cost of plant establishment or persistence and has the potential to improve human health and wellbeing and connect residents with nature [78].

Recently, South et al. [79] found the greening of vacant urban land, which includes the cleaning and greening of vacant lots via a standard, reproducible process of removing trash and debris, grading the land, planting new grass and a small number of trees, installing a low wooden perimeter fence with openings and performing regular maintenance, reduce self-reported poor mental health in community dwelling adults. In reference with the United Nations’ Sustainable Development Goals, “Goal 11: Make cities inclusive, safe, resilient and sustainable”, UGS harmonises this by augmenting urban productivity, social development and liveability—directly affecting people and their ability to advance socially and economically. Consequentially, the cyclic relationship socioeconomics and sustainable practices live up to is reflective of societal advancement and willingness to pay attention to the quality of urban development and the environment. A multiplicity of sustainability initiatives that are key to this advancement include: poverty reduction, social capital formation, good governance processes and partnerships, effective planning and management and equitable distribution of resources [80]. As a result, advanced societies are more inclined to consider these initiatives when considering and developing UGS-oriented city practices.

## 4. Conclusions

Provisional designs of modern compact cities reported positive integration of UGS due to socio-perception and -behavioural attributes by green space users [81,82]. Much of the data relates to the dose-response conceptual framework that unravels the intricacies between UGS and health [83,84]. We deem this framework as a positive, forward thinking pathway for the modernisation of smart, compact cities throughout the Asia-Pacific region and beyond.

Cities can be compact as well as ‘green,’ with meticulous attention paid to every aspect of the urban greening complex [85]. Urban planners, landscape architects and policy makers need to pay more attention to the quality of UGS and not only to the quantity [17]. Daily, people need to be in contact with nature; UGS can supply this need. For urban inhabitants, UGS is often the only source of nature-based interaction readily available within any reasonable distance; hence, the question of how much greenery a person needs is very relevant. The determined minimums by the World Health Organization conclude that, at a societal level, urban dwellers are happier and healthier when those minimums are exceeded [36]. This paper, as well as previous literature, indicates that planning practices for densification and the creation of compact cities needs to permit inclusion of UGS by way of close proximity, coherent design and sufficient size, variation, maintenance and person-to-person engagement (e.g., gardening or participatory processes) [86]. There has been recent talk of revitalising Ruskin [87] and Howard’s [88] dream of garden city living; this can be done if ecologists, landscape planners and designers smartly and attractively design high-density urban environments to include high-quality, biodiverse green space and achieve multiple functions for both people and wildlife [89]. Our underlying theme has been to develop and maintain ecologically resilient urbanisation in correlation with its rapid development. This premise has steered us toward understanding the modern compact city and, specifically, the ‘city in a garden’ approach; future best practices will need to ask how UGI requirements are being met and what UGS requirements are needed by planners and designers alike when considering future city designs. We have touched upon a variety of novel approaches and stress the importance of further research and expertise within this interdisciplinary field. Intrinsically, cities are multi-dimensional in character, rather than single. They contain the intersection of interacting components and interdependent parts of urban development and the informal urban activities that influence urban infrastructure provision and services. These provisions and services are a part of a cyclic socioeconomic relationship in which sustainability-oriented thinking is future-oriented. Such multiple interdependencies to developing UGS is a topic of further research, as it examines potential advancement of welfare and wellbeing of city dwellers as well as the adaptability for future generations. Our hope is to expand knowledge-base and work toward modernising compact cities in a sound, sustainable and vibrant manner.

## Figures and Tables

**Table 1:** Types of urban space and their effect and social costs due to poorly designed UGBS (urban green and blue space) [28].

Type of Urban Space	Effect and Social Costs
Neglected	neglecting public space, both physically and in the face of market forces
Invaded	sacrificing public space to the needs of cars, effectively allowing movement needs to usurp social ones
Exclusionary	allowing physical and psychological barriers (fear of “the other”) to dominate public space design and management strategies
Consumption	failing to address the relentless commodification of public space
Privatised	allowing public space to be privatised, with knock-on impacts on political debate and social exclusion
Segregated	reflecting the desire of affluent groups in many societies to separate themselves from the rest of society, reflecting a fear of crime or simply the desire to be exclusive
Insular	failing to halt a more general retreat from public space into domestic and virtual realms
Invented	condoning the spread of a placeless, formula-driven entertainment space
Scary	where crime and more often fear of crime, are allowed to dominate the design management and perceptions of place
Homogenised	generally presiding over a homogenisation of the public built environment in the face

**Table 2:** Compact cities site-specific performance benchmarks and design strategies and measures.

Strategies	Measures	References
Enhance visual connections	enlarge window size increase window-to-wall ratio of the courtyard walls provide visual buffers (shrubbery, for instance) for high-rises design façade/windows appropriately to facilitate visual contact with courtyard gardens below set back of the courtyard boundary (steps open to sky) use glass handrails instead of opaque parapets	Jim & Chen, 2006 [26]; Lau & Yang, 2009 [64]; Zhang & Lin, 2011 [66]
Manipulating space Morphology	control aspect ratio (D/H) to evoke a pleasant sense of space (where D = width of the space; H = height of the building flanking the space)	Lau & Yang, 2009 [64]
Facilitate natural ventilation and day lighting	modify building layout for prevailing winds create openings at proper locations as inlets and outlets for effective cross-ventilation sun path and shading study	Berkovic et al., 2012 [67]; Lau & Yang, 2009 [64]; Wania et al., 2012 [68]
Select plant species	select plants that will thrive in the climate and conditions of the site avoid invasive species that may jeopardise local ecosystems use native species use tree canopies to lessen the scale of the urban surroundings use vertical greenery/water curtain to soften hard boundaries of courtyard gardens select trees with tall trunks and relatively-narrow spreading canopies provide a green prospect from the upper windows of nearby high-rise structures if turf grasses are to be used, select them to be regionally appropriate and minimise post-establishment requirements for irrigation, pesticide, fertilizer and maintenance use medicinal plants, if applicable use edible plants avoid high BVOC emitter species along areas with heavy traffic select allergy-friendly plants	Bigirimana et al., 2012 [71]; Lau & Yang, 2009 [64]; Russo et al., 2017 [13]; SITES, 2016 [56]
Integrating vegetation in buildings	use green roof, green walls, edible green walls	Li & Yeung, 2014 [72]; SITES, 2016 [56]; Whittinghill & Rowe, 2012 [69]; Russo et al., 2017 [13]
Reduce urban heat island effects	select strategies, materials and landscaping techniques that reduce heat absorption by exterior surfaces reduce use of constructed impervious surfaces (e.g., roads, sidewalks and parking lots) increase use of vegetated surfaces and planted areas use shade from appropriate trees, large shrubs, vegetated trellises, walls or other exterior structures consider the use of new coatings and integral colorants for asphalt pavement to achieve light-coloured surfaces instead of traditional dark surface materials	Ren et al., 2013 [73]; SITES, 2016 [56]; Taleb & Abu-Hijleh, 2013 [70]

BVOC: Biogenic Volatile Organic Compounds.
